# The potential utility of acetyltanshinone IIA in the treatment of HER2-overexpressed breast cancer: Induction of cancer cell death by targeting apoptotic and metabolic signaling pathways

**DOI:** 10.18632/oncotarget.4156

**Published:** 2015-05-28

**Authors:** Mounia Guerram, Zhen-Zhou Jiang, Bashir Alsiddig Yousef, Aida Mejda Hamdi, Hozeifa Mohamed Hassan, Zi-Qiao Yuan, Hou-Wei Luo, Xiong Zhu, Lu-Yong Zhang

**Affiliations:** ^1^ Jiangsu Key Laboratory of Drug Screening, China Pharmaceutical University, Nanjing 210009, China; ^2^ Jiangsu Center for Pharmacodynamics Research and Evaluation, China Pharmaceutical University, Nanjing 210009, China; ^3^ Department of Natural Medicinal Chemistry, China Pharmaceutical University, Nanjing 210009, China; ^4^ Medical and Chemical Institute, China Pharmaceutical University, Nanjing 210009, China; ^5^ State Key Laboratory of Natural Medicines, China Pharmaceutical University, Nanjing 210009, China

**Keywords:** ATA, breast cancer, metabolism, HER2, signaling pathways

## Abstract

Increased lipogenesis and protein synthesis is a hallmark of cancer cell proliferation, survival, and metastatic progression and is under intense investigation as a potential antineoplastic target. Acetyltanshinone IIA (ATA) is a compound that was obtained from chemical modifications of tanshinone IIA (TIIA), a potent anticancer agent extracted from the dried roots of the Chinese herbal medicine *Salvia miltiorrhiza* Bunge. A previous investigation indicated that ATA is more effective in inhibiting the growth of breast cancer especially cells with HER2 overexpression. However, the molecular mechanism(s) mediating this cytotoxic effect on HER2-positive breast cancer remained undefined. Studies described here report that ATA induced G1/S phase arrest and apoptosis in the HER2-positive MDA-MB-453, SK-BR-3, and BT-474 breast cancer cell lines. Mechanistic investigations revealed that the ATA-induced apoptosis effect is associated with remarkably down-regulation of receptor tyrosine kinases (RTKs) EGFR/HER2 and inhibition of their downstream pro-survival signaling pathways. Interestingly, ATA was found to trigger oxidative and endoplasmic reticulum (ER) stresses and to activate AMP activated protein kinase (AMPK) leading to inactivation of key enzymes involved in lipid and protein biogenesis. Intraperitoneal administration of ATA significantly inhibited the growth of MDA-MB-453 xenografts in athymic mice without causing weight loss and any other side effects. Additionally, transwell migration, invasion, and wound healing assays revealed that ATA could suppress tumor angiogenesis *in vitro*. Taken together, our data suggest that ATA may have broad utility in the treatment of HER2-overexpressed breast cancers.

## INTRODUCTION

Breast cancer is by far the most common non-cutaneous malignancy affecting women worldwide. Despite considerable progress in both diagnosis and treatment, breast cancer remains the second leading cause of cancer-related death in women [1, 2]. The importance of the human epidermal growth factor receptor (HER, also known as ErbB) family in the development and progression of various cancers is widely recognized [3]. Family members include four receptors: HER1 (EGFR/ErbB1), HER2 (ErbB2), HER3 (ErbB3), and HER4 (ErbB4) [4]. Each has been reported to be amplified or overexpressed in some forms of breast cancer, with HER2 and EGFR being the most extensively studied. Overexpression of HER2, found in more than 30% of breast cancer cases [5, 6], is associated with poor prognosis and considered as a predictive marker of chemoresistance. Thus, therapeutic strategies focusing on the inhibition of this oncogene are now being actively explored in breast cancer therapy.

Cancer metabolic reprogramming, such as enhanced glycolysis and lipid biosynthesis, has been recognized as one of the important characteristic features of cancer [7, 8]. Increased lipogenesis, found in approximately 20% to 90% of cancer cases, is a hallmark of aggressive cancers and is reflected in the up-regulation of key enzymes involved in this multiple step process [9]. These include fatty acid synthase (FASN), acetyl-CoA carboxylase (ACC) and ATP-citrate lyase (ACLY). Several studies reported that these lipogenic enzymes are overexpressed in a number of human malignancies including breast cancer [9–14]. Interestingly, their inhibitions by specific inhibitors or siRNA caused significant growth suppression and induced apoptosis in different cancer models [15–20]. New and effective therapeutic agents and strategies targeting these key biosynthetic enzymes are now being actively explored in anticancer therapies [9, 15, 17, 21–24]. Importantly, recent investigations suggest a cross-talk between these metabolic enzymes and the human epidermal growth factor receptor family, especially HER2, via different signaling pathways [12, 25, 26].

**Figure 1:**
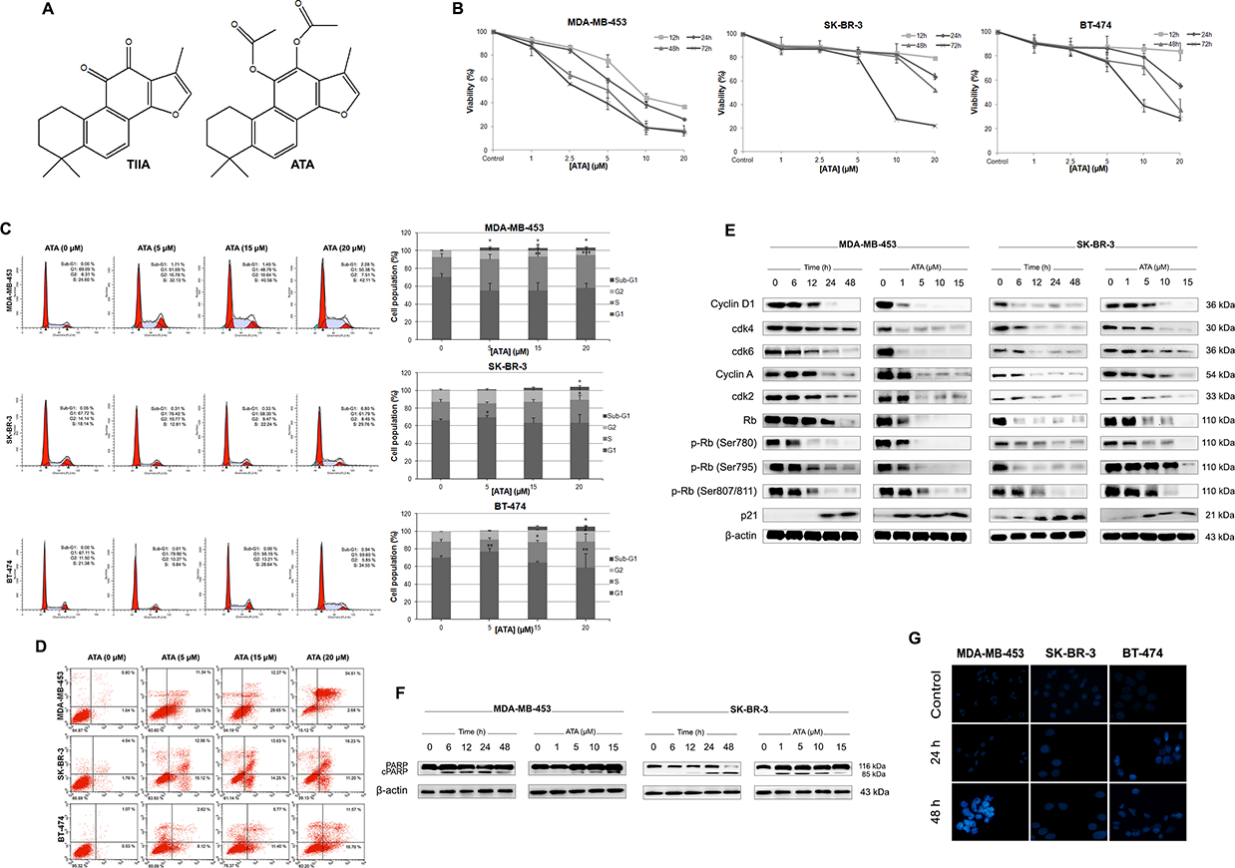
Induction of cell cycle arrest and apoptosis by ATA in HER2-positive breast cancer cells **A.** Chemical structures of acetyltanshinone IIA (ATA) and tanshinone IIA (TIIA). **B.** Effect of ATA on the cell viability of MDA-MB-453, SK-BR-3, and BT-474. Cells were incubated with ATA at various concentrations (1 μM, 2.5 μM, 5 μM, 10 μM, and 20 μM) and for different periods of time (12, 24, 48 and 72 h). Cell viability was measured by CCK-8 assay. **C.** ATA induced G1/S phase arrest. Following treatment with ATA (5 μM, 15 μM, and 20 μM) for 48 h, cells were analyzed for PI-stained DNA content by flow cytometry. Graphs represent different phases of the cell cycle with each bar representing the mean ± SD of data from three independent experiments. *p < 0.05, **p < 0.01, ***p < 0.001 versus control. **D.** ATA induced apoptosis in HER2-positive breast cancer cells. Treated and untreated cells were harvested, washed with PBS, stained with Annexin V-FITC and PI, and analyzed for Annexin V/PI positivity by flow cytometry. **E.** Cellular levels of key regulators of cell cycle: cyclin D1, cyclin A, cdk2, cdk4, cdk6, Rb, p-Rb (Ser 780), p-Rb (Ser 795), p-Rb (Ser 807/811), and p21 were analyzed by Western blotting. β-actin was used as loading control. **F.** Immunoblotting analysis of apoptosis-related protein, PARP. β-actin was used as loading control. **G.** Nuclear morphological changes induced by ATA in breast cancer cells. Following treatment with ATA (10 μM) for 24 h and 48 h, nuclei were stained with Hoechst 33258 (blue) and observed under fluorescence microscope (magnification, 200×).

Natural products derived from plants are major resources of prospective chemopreventive and chemotherapeutic candidates [27, 28]. Tanshinone IIA is one of the most active components found in the dried roots of *Salvia miltiorrhiza* Bunge (also known as Danshen). Previous studies indicated that tanshinone IIA possesses potent anti-inflammatory [29] and growth inhibitory properties against different cancer types [30–35]. Acetyltanshinone IIA (ATA) is a novel compound that was chemically modified from tanshinone IIA (Fig. 1A) and has been identified as a promising agent against breast cancer [36]. The current study show the potent anti-cancer activity of ATA on HER2-overexpressing breast cancer cells and explore its *in vivo* therapeutic potential in a mouse xenograft model of HER2-amplified breast cancer. Furthermore, mechanistic investigations described here reveal a novel anticancer mechanism of ATA via targeting different oncogenic signaling pathways.

## RESULTS

### ATA inhibited the viability of HER2-overexpressing human breast cancer cells

Initially we investigated the cytotoxic effect of ATA on the proliferation of a panel of HER2-overexpressing breast cancer cell lines including MDA-MB-453, SK-BR-3, and BT-474 cells. We found that ATA caused a significant time- and dose-dependent reduction in the viability of all tested cell lines (Fig. 1B) with a 50% inhibitory concentration (IC_50_) ranging between 2 μM and 9 μM as determined by CCK-8 assay (Table 1). To ascertain whether ATA had any selectivity for normal versus cancer cells, human normal breast epithelial cells MCF-10A was treated with ATA. We found that the IC_50_ value for this normal breast cell line is significantly higher than the IC_50_ values of ATA on breast cancer cells (Table 1). Thus, ATA was more able to selectively inhibit the growth of breast cancer cells than non-cancer ones.

**Table 1:** Antiproliferative activity data of ATA against human breast cancer and non-cancer cell lines

Cell lines	Origin	Immunoprofile	ATA IC_50_ (μM)^a^
MDA-MB-453	Breast carcinoma	ER -, PR -, HER2 +	1.97 ± 1.16
SK-BR-3	Breast carcinoma	ER -, PR -, HER2 +	9.17 ± 0.42
BT-474	Breast carcinoma	ER +, PR +/−, HER2 +	6.79 ± 1.40
MCF-10A	Non-cancer breast cell line	ER -, PR -, HER2 -	31.74 ± 4.24

a Values, expressed as IC_50_ (50% growth inhibition), are given in micromolar (μM) and are mean ± SD of three independent experiments.

### ATA mediated inhibition of cancer cell growth by inducing cell cycle arrest and apoptosis

To determine the cause and the nature of cell death by which ATA mediated its cytotoxic effects on HER2-positive breast cancer cells, cell cycle progression and apoptosis were examined by flow cytometry. Data revealed that treatment with ATA caused S phase cell cycle arrest in MDA-MB-453 cells whereas SK-BR-3 and BT-474 cells were arrested at G1 phase when treated with the low dose of 5 μM and S phase when treated with the high dose of 20 μM. The representative FACS histograms are shown in Fig. 1C. Additionally, a dose-dependent increase in the sub-G1 and Annexin V-positive cell populations observed in ATA-treated groups indicate an induction of apoptosis (Fig. 1C and 1D).

To examine the molecular alterations associated with these events, expression levels of several cell cycle and apoptosis-related proteins were determined by Western blot analysis. As expected, cellular levels of G1-S phases proteins: Cyclin D1, cdk4 and 6 (G0/G1 phase); and Cyclin A, cdk2 (S phase) were markedly down-regulated following ATA treatment (Fig. 1E). Moreover, our data indicate that ATA resulted in an increase of cyclin-dependent kinase inhibitor p21 and a decrease of retinoblastoma (Rb) phosphorylation at serine 780, serine 795, and serine 807/811 (Fig. 1E). *Poly (ADP-ribose) polymerase* (PARP), an early marker of apoptosis, was found to be cleaved dose- and time-dependently following ATA exposure (Fig. 1F). Additionally, morphological changes demonstrated by Hoechst 33258 staining confirmed the induction of apoptosis (Fig. 1G). Together, our results clearly indicate that the anticancer activity of ATA on breast cancer cells is associated with induction of cell cycle arrest and apoptosis and that this action is not only time- and dose-dependent but also cell line-specific.

### ATA depleted EGFR/HER2 levels and down-regulated multiple intracellular signaling pathways

**Figure 2:**
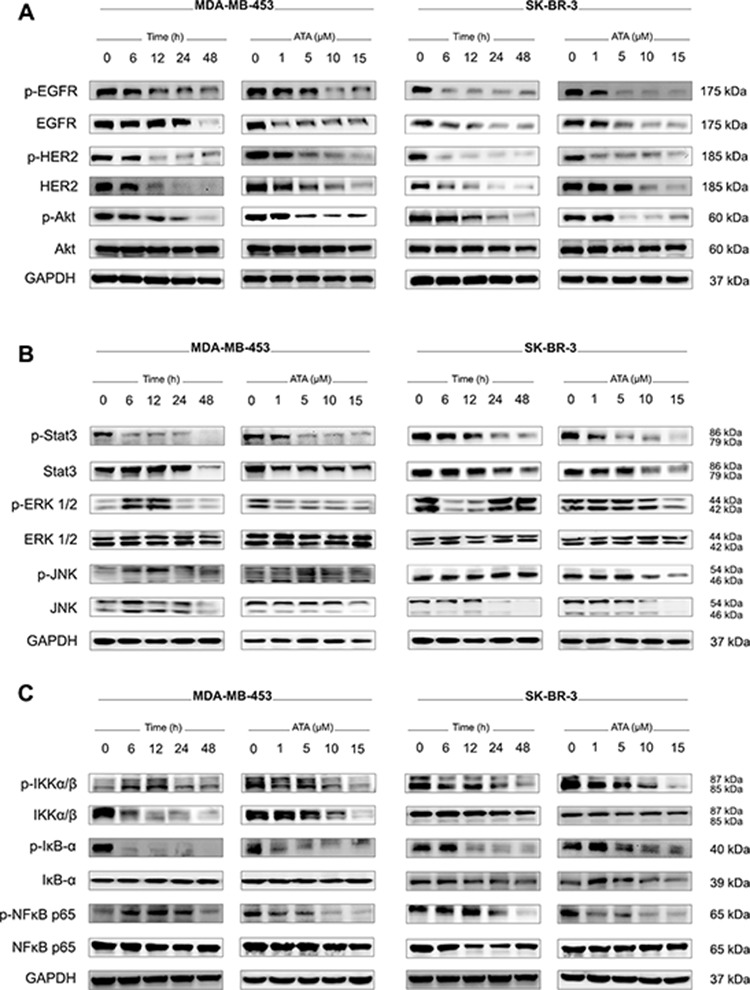
ATA down-regulates EGFR/HER2 cellular levels and inhibits downstream pro-survival signaling pathways **A.** Representative Western blot analysis of p-EGFR, total EGFR, p-HER2, total HER2, p-Akt, and total Akt expressed in MDA-MB-453 and SK-BR-3 cells treated with ATA. GAPDH was used as loading control. **B.** Representative Western blot analysis of p-Stat3, total Stat3, p-ERK1/2, total ERK1/2, p-JNK, and total JNK expressed in MDA-MB-453 and SK-BR-3 cells treated with ATA. GAPDH was used as loading control. **C.** Representative Western blot analysis of p-IKKα/β, total IKKα/β, p-IκB-α, total IκB-α, p-NFκB p65, and total NFκB p65 expressed in MDA-MB-453 and SK-BR-3 cells treated with ATA. GAPDH was used as loading control.

EGFR and HER2 expression levels were evaluated in MDA-MB-453 and SK-BR-3 treated-cells. Results indicate that ATA induced a down-regulation of the cellular levels of HER2 and its dimerization partner EGFR (Fig. 2A). The phosphorylation/activation status of these oncoproteins was also strongly reduced in a dose- and time-dependent manner (Fig. 2A). Next, we analyzed the effect of ATA on signaling pathways usually activated by these receptor tyrosine kinases (RTKs). Western blotting data revealed a down-regulation of the phosphorylation status of Akt, Stat3, and NFκB p65 (Fig. 2A, 2B, and 2C). In some instances, the reduced phosphorylation was accompanied by concomitant reduction of total protein amounts (Fig. 2A, 2B, and 2C).

### ATA induced activation of AMPK and altered the expression levels of key enzymes involved in lipid and protein biosyntheses

**Figure 3:**
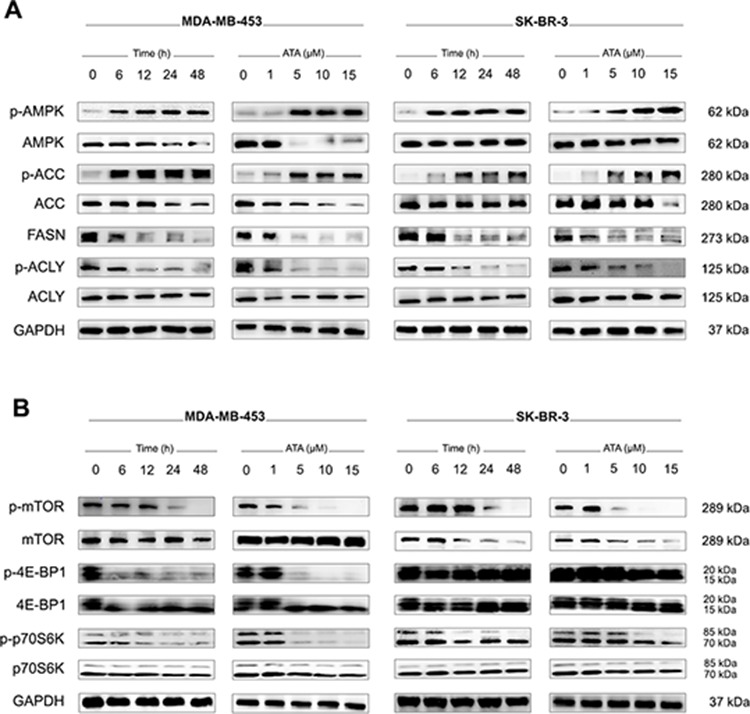
Western blotting for the effect of ATA on the signals associated with AMPK/ACC/FASN/ACLY and mTOR/4E-BP1/p70S6K pathways **A.** Whole cell lysates were prepared and Western blot analysis was conducted using anti-p-AMPK, -AMPK, -p-ACC, -ACC, -FASN, -p-ACLY, and -ACLY antibodies. GAPDH was used as loading control. **B.** Representative Western blot analysis of p-mTOR, total mTOR, p-4E-BP1, total 4E-BP1, p-p70S6K, and total p70S6K expressed in MDA-MB-453 and SK-BR-3 cells treated with ATA. GAPDH was used as loading control.

Recent studies reported that inhibition of fatty acid synthesis can suppress tumor cell growth and induce apoptosis [15, 17, 18, 21, 37–42]. This inhibition can be achieved through impedance of a variety of enzymatic steps in the pathway. In the current study, expression levels of three key lipid biosynthetic enzymes (ACLY, FASN, ACC) were verified by Western blot. As shown in Fig. 3A, treatment with ATA resulted in a decrease of the protein levels of FASN and p-ACLY compared to those in DMSO-treated control cells. No change in total ACLY was observed. Simultaneously, cellular levels of p-ACC significantly increased following ATA exposure (Fig. 3A). Moreover, evaluation of the cellular level of AMPK, a metabolic sensor of cellular energy state, indicated that ATA induced the activation of AMPK with gradually increased levels of p-AMPK (Fig. 3A) whereas the cellular levels of p-mTOR and its downstream targets p-p70S6K and p-4E-BP1, key enzymes involved in protein synthesis, decreased (Fig. 3B). It seems that ATA inactivates mTOR via concomitant activation of AMPK and inhibition of Akt, thereby causing cell cycle arrest and apoptosis.

### ATA triggered ER and oxidative stresses in HER2-positive breast cancer cells

**Figure 4:**
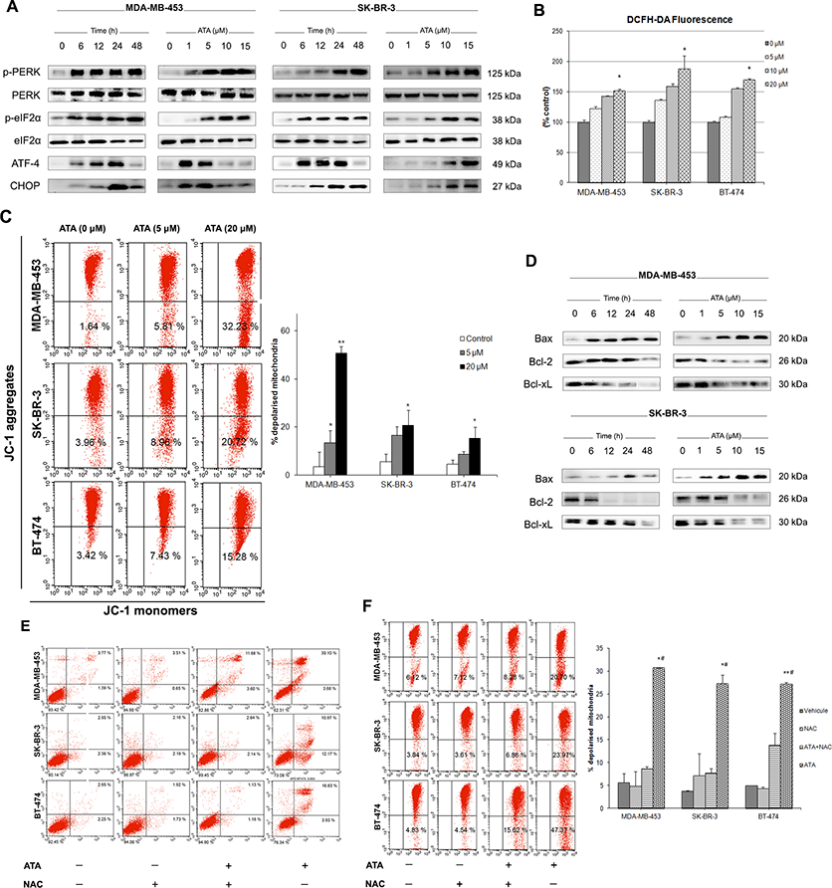
ATA triggers ER and oxidative stresses and induces loss of mitochondrial membrane potential **A.** Representative Western blot analysis of p-PERK, total PERK, p-eIF2α, total eIF2α, ATF-4, and CHOP expressed in MDA-MB-453 and SK-BR-3 cells following ATA treatment. **B.** Effect of ATA on ROS generation in breast cancer cells. Cultured cells labeled with DCFH-DA were incubated in the presence or absence of ATA (5 μM, 10 μM, and 20 μM). Fluorescence signal was monitored time-dependently using a fluorescent plate reader. Each bar represents mean ± SD and is plotted for sextuplicate from each condition (*p < 0.05 versus control). **C.** ATA induced mitochondrial membrane depolarization. Graphs represent percentages of breast cancer cells with depolarized mitochondria as determined by flow cytometry. Data are presented as mean ± SD from three independent experiments (*p < 0.05, **p < 0.01 versus control). **D.** Representative Western blot analysis of Bax, Bcl-2, and Bcl-xL expressed in MDA-MB-453 and SK-BR-3 cells treated with ATA. **E and F.** NAC prevented ATA-induced apoptosis and loss of MMP. Breast cancer cells were pre-incubated for 2 h in the presence or absence of NAC, and then treated with ATA for 48 h. (E) Induction of apoptosis was determined by flow cytometry. (F) Loss of MPP was assessed using JC-1 assay kit. Percentages of breast cancer cells with depolarized mitochondria are plotted as mean ± SD (*n* = 3). *p < 0.05, **p < 0.01 versus control, #*p* < 0.05 compared to ATA+NAC group.

In eukaryotic cells, endoplasmic reticulum (ER) is an important player in regulating protein synthesis and lipid metabolism [43]. Perturbation of ER homeostasis is known as ER stress [44]. To assess whether ATA can cause ER stress in MDA-MB-453 and SK-BR-3 cells, we examined by Western blot analysis the phosphorylation status of the ER stress sensor, PERK. We found that ATA induced phosphorylation/activation of PERK which, in turn, phosphorylated/activated the translation initiation factor eIF2α (Fig. 4A). Transcription factor ATF4, a downstream signal of PERK-eIF2α pathway, was also found to be up-regulated. In addition, expression of CHOP, a hallmark of ER stress-mediated cell cycle arrest and apoptosis [45], was increased (Fig. 4A). Coupled with the phosphorylation of PERK, increases in steady-state levels of the pro-apoptotic proteins ATF-4 and CHOP with simultaneous decrease in general protein synthesis strongly indicate that the induction of ER stress might be involved in ATA-mediated cell death in HER2-positive breast cancer cells.

Accumulation of reactive oxygen species (ROS) in the cytoplasm has been shown to trigger ER stress and the unfolded protein response [46] leading to CHOP expression [47], cleavage of PARP, and apoptosis [48]. We used the fluorescent probe DCFH-DA to monitor intracellular ROS levels in the presence and absence of ATA. We found that ATA caused an early and slight, but statistically significant, increase in ROS accumulation (Fig. 4B). Furthermore, FACS analysis using JC-1 dye indicated a significant increase (*p* < 0.05) in the percentage of cells with depolarized mitochondria in ATA-treated cells compared to DMSO-treated control (Fig. 4C). Additionally, ATA notably increased the levels of proapoptotic protein Bax and concomitantly decreased the expression of antiapoptotic proteins Bcl-2 and Bcl-xL (Fig. 4D).

To determine whether increased production of ROS may have a role in ATA-induced apoptosis, loss of mitochondrial membrane potential (MMP), or cell cycle arrest; HER2-positive breast cancer cells were pretreated with the antioxidant *N*-acetyl-*L*-cysteine (NAC) two hours before adding ATA for a further 48 h. As shown in Fig. 4E and 4F, pretreatment with NAC conferred significant protection against ATA-induced apoptosis and loss of MMP. However, the same treatment did not prevent cell cycle arrest (data not shown). Together, these data indicate that ROS generation may be one of the important events in the ATA-activated apoptotic signaling cascade.

### ATA inhibited tumor growth *in vivo*

**Figure 5:**
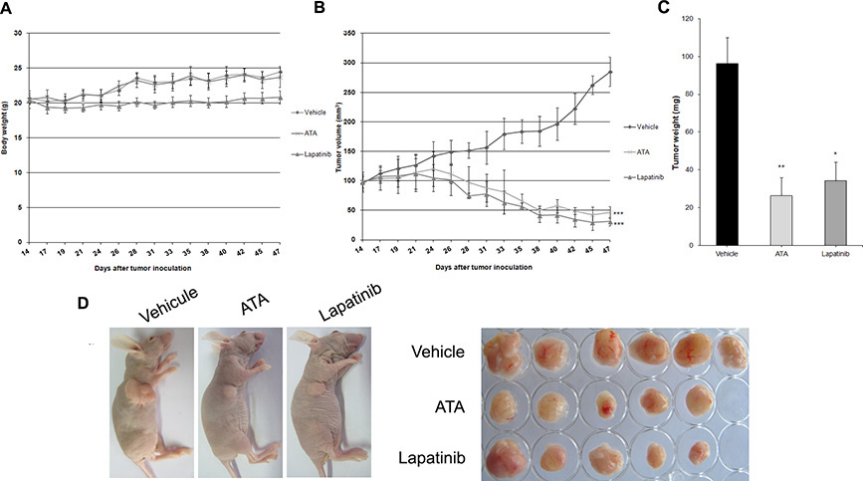
Effect of ATA on MDA-MB-453 xenograft tumor growth MDA-MB-453 cells (2 × 10^7^ per mouse) were implanted subcutaneously into the right flank of BALB/c nude mice. After solid tumors grew, mice were randomly divided into three groups (*n* = 6) and treated intraperitoneally with vehicle (negative control group), or ATA (35 mg/kg). Positive control group received lapatinib (80 mg/kg) via gastric gavage twice daily. ATA inhibited tumor growth as measured by tumor volume **B.** and tumor weight **C.** without side effects as shown by mice body weight **A.** Data are presented as mean ± SD. *p < 0.05, **p < 0.01, ***p < 0.001 versus control. **D.** At the end of the treatment-period, mice were sacrificed and tumors were excised.

We used a xenograft model of HER2-positive MDA-MB-453 breast cancer in nude mice to explore the *in vivo* therapeutic potential of ATA. We found that 35 mg/kg of ATA administered three times per week significantly reduced tumor volume (Fig. 5B) and tumor weight (Fig. 5C) but had no effect on mice body weight (Fig. 5A). In this tumor inhibition model, the average tumor volume in the negative control group increased from 95.79 ± 12.02 mm^3^ to 285.27 ± 25.24 mm^3^ at the end of the experiment, whereas the average tumor volume in the ATA-treated group decreased from 99.55 ± 11.13 mm^3^ to 46.49 ± 10.20 mm^3^. Tumor regression occurred in ATA-treated group by day 14 (~2%) after starting treatment and reached ~50% on day 33 (end-point of the experiment). Additionally, the average tumor weight in the negative control group was 96.33 ± 13.67 mg, whereas the average tumor weight in the ATA-treated group was only 26.5 ± 9.42 mg (Fig. 5C) suggesting that ATA strongly inhibited tumor growth *in vivo*.

### ATA suppressed the motility of HER2-overexpressing breast cancer cells and inhibited angiogenesis *in vitro*

**Figure 6:**
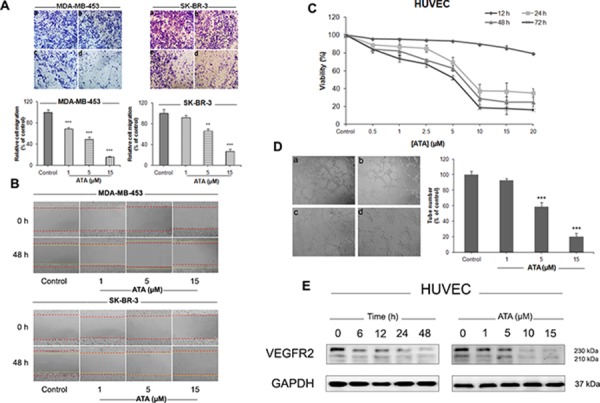
ATA supresses the motility of HER2-positive breast cancer cells and inhibits HUVECs proliferation and tubular formation **A.** ATA inhibited MDA-MB-453 and SK-BR-3 cells migration in a transwell assay. Representative images and summary data (bar graph on bottom side) indicate the inhibition of migration. Cells were treated for 48 h with vehicle (a), 1 μM ATA (b), 5 μM ATA (c), or 15 μM ATA (d). Magnification: 100×. The results are expressed as mean ± SD (*n* = 3); **p < 0.01, ***p < 0.001 versus control. **B.** ATA treatment inhibited cell motility in a wound-healing test. Representative images before and after incubation with vehicle or ATA (1 μM, 5 μM, and 15 μM) (red lines indicate time 0 h and yellow lines indicate time 48 h). Magnification: 100×. **C.** Effect of ATA on the viability of HUVECs. Cells were exposed to different concentrations of ATA as indicated for 12, 24, 48 and 72 h. At the end of each time-period, cell viability was measured by CCK-8 assay. **D.** ATA inhibited HUVECs tube formation. Representative images and summary data (bar graph on right hand side) indicate the inhibition of tube formation. HUVECs were treated with vehicle (a), 1 μM ATA (b), 5 μM ATA (c), or 15 μM ATA (d) for 6 h. Magnification: 100×. The results are expressed as mean ± SD (*n* = 3); ***p < 0.001 versus control. **E.** Representative Western blot analysis of VEGFR-2 expressed in HUVECs treated with ATA. GAPDH was used as loading control.

It is a well-known fact that cell migration and invasion plays a crucial role in tumor metastasis. In the present study, wound-closure and transwell migration assays were employed to assess the effects of ATA on the motility of HER2-positive breast cancer cells. Results shown in Fig. 6A and 6B indicate an apparent antimobility effect of ATA as illustrated with MDA-MB-453 and SK-BR-3 cells compared to their respective negative controls.

We further investigated the effect of ATA on the proliferation, migration, invasion, and tube formation of human umbilical vascular endothelial cells (HUVECs). As depicted in Fig. 6C, ATA resulted in a dramatic inhibition of HUVE cell proliferation. Capillary-like tube formation assay indicates that ATA exposure markedly and dose-dependently suppressed the formation of functional tube-like networks (Fig. 6D). Furthermore, ATA inhibited the chemotactic motility and invasion of HUVECs as revealed by wound-healing and transwell techniques (data not shown). To investigate the molecular mechanism underlying this anti-angiogenic effect, we evaluated the cellular levels of vascular endothelial growth factor receptor-2 (VEGFR2) by Western blot analysis. As shown in Fig. 6E, ATA time- and dose-dependently down-regulated the protein expression of VEGFR2 in HUVECs. Cumulatively, these data suggest that ATA may inhibit angiogenesis *in vitro*.

## DISCUSSION

The development of innovative ways to prevent and/or to treat resistant subtypes of breast cancer is being actively explored in cancer chemotherapy. We focused our investigation on acetyltanshinone IIA (ATA) which is described as a promising cytotoxic agent from herbal origin with potent *in vitro* and *in vivo* anticancer properties [36]. We explored for the first time the molecular mechanisms by which ATA inhibits angiogenesis, metastasis, and induces cell death in HER2-overexpressing breast cancer cells. We further examined the intricate relationship between ATA-induced apoptosis and inhibition of key metabolic enzymes involved in lipid and protein biogenesis.

Data reported here show that ATA inhibited dose- and time-dependently the proliferation of HER2-positive MDA-MB-453, SK-BR-3, and BT-474 cell lines. More importantly, when we investigated whether ATA has a selective activity on cancer cells, we found that its action is relatively less potent on MCF-10A, a non-tumorigenic breast cell line (Table 1). These results enhance the desirability of ATA as an anticancer agent because they suggest that it selectively kills breast cancer cells while minimizing damage to normal breast tissue.

FACS and Western blot analyses revealed that the anticancer activity of ATA is associated with cell cycle arrest and apoptosis. Perturbations of cell cycle were mainly found in the increase of G1 and S phases cell populations. Further investigations on the status of key factors known to regulate cell cycle progression indicated that treatment with ATA promoted the levels of cyclin-dependent kinase inhibitor p21 and down-regulated the protein expressions of cyclin D1, cyclin A, cdk2, cdk4, and cdk6. This action prevented cyclin D/cdk4/cdk6 and cyclin A/cdk2 complexes from phosphorylating Rb protein at different serine positions thereby arresting cell growth. Moreover, observations that ATA induced S phase arrest in MDA-MB-453 and G1 and S phases arrest in SK-BR-3 and BT-474 depending on the dose used for treatment suggest that ATA-mediated growth inhibition may be cell specific.

Cancers overexpressing receptor tyrosine kinases (RTKs) EGFR and/or HER2, particularly breast cancers [49], are known to be resistant to established chemotherapy [50]. Thus, dual kinase inhibition directed against EGFR and HER2 was and still is an attractive potential approach for breast cancer therapy. Our data showed a decrease in the phosphorylation/activation status of HER2 and EGFR oncoproteins following ATA exposure which consecutively inhibited the activation of downstream pro-survival growth pathways.

The survival of breast cancer cells with HER2 gene amplification has been reported to be heavily dependent on lipid metabolism [51]. Several studies reported a crosstalk between FASN and HER2 oncoprotein [52–55]. Indeed, HER2 overexpression was found to increase translation of FASN [12, 56], whereas FASN overexpression was found to markedly increase EGFR and HER2 signalings [25]. Furthermore, tumor cells are well-characterized by their rapid cell growth which requires active synthesis of proteins, ribosomal RNA, and lipids. As all of these functions are switched off by AMPK [57, 58], tumor cells developed mechanisms to down-regulate AMPK and thus escape from its restraining influences on growth and biosynthesis. Herein, we have shown that treatment with ATA induced phosphorylation/activation of AMPK and that this effect translated to downstream targets such as phosphorylation/inhibition of ACC, down-regulation of FASN and p-ACLY, and inhibition of mTOR/4EB-P1/p70S6K signaling pathway. Interestingly, we found that ATA triggered ER and oxidative stresses, induced loss of MMP, and altered protein expression of Bcl-2, Bcl-xL, and Bax in HER2-positive breast cancer cells. More important, employing the powerful antioxidant NAC [59], we demonstrated that ROS play a critical role in ATA-induced apoptosis and loss of MMP.

Overexpression of HER2 in human tumor cells is closely associated with increased angiogenesis and expression of vascular endothelial growth factor (VEGF) [60]. Proliferation, migration, and tube formation of endothelial cells represent the three primary steps of angiogenesis [61] and play a central role in tumor growth, metastasis, and survival. In the present study, we provided evidence that ATA significantly and dose-dependently inhibits the mobility of HER2-positive breast cancer cells. We further showed that endothelial cells (HUVECs) proliferation, migration, invasion, and tubule formation are markedly disrupted following ATA exposure. Consistent with these results, VEGFR2 protein expression was found to be down-regulated (Fig. 6E). As VEGFR2 is the main receptor involved in endothelial cell survival, proliferation, and vascular permeability; VEGFR2 suppression may represent a critical mechanism by which ATA mediates its anti-angiogenic effects.

To the best of our knowledge, this is the first study demonstrating the detailed molecular mechanism of ATA on HER2-overexpressing breast cancer cells. Altogether our findings described the ability of ATA to perturb four major cellular signaling and metabolic pathways: HER2/EGFR, ROS/ER stress, proteins and lipids biogenesis. On the basis of these promising results, the present study suggests that ATA is a potential option for the treatment of patients with HER2-driven breast cancer.

## MATERIALS AND METHODS

### Drugs and reagents

ATA (MW 380 g/mol) was provided by China Pharmaceutical University Research Institute of Pharmaceutical Chemistry. It was obtained as a white powder of high purity (> 97%). Stock solutions of 10 mM were dissolved in dimethyl sulfoxide (DMSO, Sigma-Aldrich), stored at − 20°C, and diluted in fresh medium just before use. Lapatinib (MW 925.3 g/mol) was obtained from Jiangsu Kanion Pharmaceutical Co., Ltd. (China). Drugs dilutions were made in serum-free media with a final DMSO concentration less than 0.1%. Media and reagents for cell culture were acquired from Thermo Scientific HyClone (Beijing, China). *N*-acetyl-*L*-cysteine (NAC) was purchased from Sigma-Aldrich (China). Antibodies employed in this study were obtained from Santa Cruz Biotechnology, Inc. (Santa Cruz, CA, USA) and Cell Signaling Technology (Beverly, MA, USA). Hoechst 33258 was from Invitrogen (CA, US). Cell Counting Kit-8 (CCK-8) was purchased from Dojindo Laboratories (Kumamoto, Japan). Annexin V-FITC Apoptosis Detection Kit was from BD Pharmingen™ (San Diego, CA, USA). Other kits were acquired from Beyotime Biotechnology Co., Ltd. (Nantong, China) and KeyGEN Biotechnology Co., Ltd. (Nanjing, China).

### Cells and culture conditions

Three human breast cancer cell lines (MDA-MB-453, SK-BR-3, and BT-474) characterized by amplification and/or overexpression of the HER2 oncogene and a normal breast epithelial cell line (MCF-10A) were used in this study. Cells were obtained from the Cell Bank of Shanghai Institute of Cell Biology, Chinese Academy of Sciences (Shanghai, China) and were grown as monolayers in the following media: RPMI 1640 (BT-474 and MCF-10A); DMEM (SK-BR-3); and Leibovitz's (MDA-MB-453). All media were supplemented with 10% (v/v) fetal bovine serum (FBS) and 1% (v/v) antibiotics. Cells were maintained at 37°C in a humidified atmosphere consisting of 5% CO2 and 95% air except MDA-MB-453 cells which were maintained in an atmosphere without CO2.

### Cell viability assay

The effect of ATA on cell viability was determined by Cell Counting Kit-8 (CCK-8) assay. Briefly, exponentially growing cells were plated at appropriate seeding density in 96-well plates and allowed to attach overnight. Next day, cells were treated with media containing DMSO alone as control or with ATA (1 μM, 2.5 μM, 5 μM, 10 μM, and 20 μM) for 12, 24, 48, and 72 h. Cell viability was determined at the end of each time-period using CCK-8 reagent. Absorbance was measured at 450 nm with a microplate reader (Bio-Rad Laboratories, Hercules, CA, USA).

### Flow cytometry

To examine the mitochondrial membrane potential (MMP) after ATA-treatment, JC-1 (5, 5′, 6, 6′-tetrachloro-1, 1′, 3, 3′-tetraethylbenzimidazolylcarbocyanine iodide) dye was used [62] according to the manufacturer's instructions. For apoptosis detection, Annexin V-FITC Apoptosis Detection Kit I (BD Biosciences Pharmingen) was employed following the manufacturer's protocol. For cell cycle analysis, treated and untreated cells were washed with phosphate buffered saline (PBS), fixed overnight in ice-cold 70% (v/v) ethanol, and stained with a staining buffer containing RNase and propidium iodide (PI). Data acquisition was done by flow cytometry (Becton Dickinson, San Jose, CA) using Cell Quest and ModFit LT softwares.

### Immunoblotting

Following ATA exposure, whole-cell lysates were isolated using KeyGEN Total Protein Extraction Kit following the manufacturer's instructions. Protein concentrations were determined by the BCA method and protein extracts were stored at −80°C until use. For Western blot analysis, equal amounts of proteins were electrophoresed on sodium dodecylsulfate polyacrylamide gel electrophoresis (SDS-PAGE) gels and transferred to nitrocellulose membranes (0.45 μm, Bio-Rad Laboratories, Hercules, CA, USA) by electroblotting. Proteins were then detected with specific antibodies. For each test, an anti-β-actin or anti-GAPDH antibody was used as loading control. Protein signals on nitrocellulose membranes were assessed with ChemiDoc XRS imaging densitometer (Bio-Rad) using Quantity One software program (Bio-Rad Laboratories, CA, USA).

### Measurement of ROS production

The intracellular ROS levels were determined spectroflurometrically using the membrane-permeable fluorescent probe 2′, 7′-dichlorodihydrofluorescein diacetate (DCFH-DA, Beyotime). Briefly, cells (2.5 × 10^4^ per well) were seeded in 96-well black wall/clear bottom microplates. After allowing cells to attach overnight, 100 μL of DCFH-DA staining solution (5 μM) was added to each well and cells were incubated in the dark at 37°C for 1 h. At the end of the incubation time, cells were washed twice with serum-free media and 100 μL of ATA dilutions (5 μM, 10 μM, and 20 μM) were added to each well (six wells per concentration). Background wells (untreated or ATA treated cells) as well as blank wells (media only) were included in each test. Fluorescence signal was then monitored time-dependently using a fluorescent plate reader (Tecan Austria Gmbh) with Magellan software. Blank readings were subtracted from all measurements and relative ROS production was expressed as fold change in fluorescence compared with the fluorescence of the corresponding control.

### Transwell migration and wound healing assays

Transwell motility assays were performed as previously described [63] with minor modifications. The chemoattractant used for these tests was complete media. Assays were performed using 8-μm transwell inserts (Millipore). Migrated cells were fixed with 4% paraformaldehyde and stained with Crystal Violet. For wound healing tests, confluent monolayers of cells were scratched with a micro pipette tip. All assays were monitored over a 48 h-period. Pictures of five random fields were captured with an Olympus IX71 microscope. All experiments were performed in triplicate.

### Capillary-like tube formation (HUVEC) assay

Growth factor-depleted Matrigel from BD Pharmingen (San Jose, CA, USA) was applied to a 96-well tissue culture plate (50 μl per well) [64]. Following polymerization, human umbilical vein endothelial cells (HUVECs) were seeded at a density of 2 × 10^4^ cells per well (final volume 180 μL) on the polymerized Matrigel in the presence or absence of ATA (1 μM, 5 μM, and 15 μM). Plate was incubated at 37°C, 5% CO2 for 6 h. Photographs of five random fields were taken under an inverted microscope and tube formation was quantified using Adobe Photoshop software. The inhibition of tube formation was expressed relative to untreated control wells which were set at 100%.

### *In vivo* study

5–6 weeks old female BALB/c nude mice weighing ~20 g each were purchased from Shanghai Rubicam Laboratory Animal Ltd (Shanghai, China) and housed in a controlled environment at 22°C with a 12 h light/dark cycle. MDA-MB-453 cells with ~92% viability were subcutaneously injected into the right flank of each mouse (2 × 10^7^ per mouse). After tumors grew to ~90 mm^3^, mice were randomly selected (6 mice/group) for the intraperitoneal administration of either ATA at a dose of 35 mg/kg or vehicle three times per week. The positive control group was treated by gastric gavage with lapatinib (80 mg/kg) twice daily. The body weight of each mouse was recorded three times a week (Monday, Wednesday, and Friday). At the same time, solid tumor volume (in cubic millimeters) was determined using digital Vernier caliper measurements and the formula of *a* × *b*^2^ × 0.5, where *a* is the longest diameter of the tumor and *b* is the shortest diameter of the tumor. At the end of the treatment period, mice were sacrificed and tumors were excised. All animal procedures were approved by the Animal Ethics Committee of China Pharmaceutical University, Nanjing University, and Laboratory Animal Management Committee of Jiangsu Province.

### Statistical analysis

Student's two-tailed *t* test was used to determine the statistical significance of difference in the measured variables between control and treated groups. For all analyses, a *p* value less than 0.05 was considered statistically significant.
